# Dysfunctional Mitochondria and Mitophagy as Drivers of Alzheimer’s Disease Pathogenesis

**DOI:** 10.3389/fnagi.2019.00311

**Published:** 2019-11-20

**Authors:** Anushka Chakravorty, Cuckoo Teresa Jetto, Ravi Manjithaya

**Affiliations:** ^1^Autophagy Laboratory, Molecular Biology and Genetics Unit, Jawaharlal Nehru Centre for Advanced Scientific Research, Bengaluru, India; ^2^Neuroscience Unit, Jawaharlal Nehru Centre for Advanced Scientific Research, Bengaluru, India

**Keywords:** mitochondrial dysfunction, mitophagy, Alzheimer’s disease, microglia, amyloid beta, tau

## Abstract

Neurons are highly specialized post-mitotic cells that are inherently dependent on mitochondria owing to their high bioenergetic demand. Mitochondrial dysfunction is therefore associated with various age-related neurodegenerative disorders such as Alzheimer’s disease (AD), wherein accumulation of damaged and dysfunctional mitochondria has been reported as an early symptom further contributing to disease progression. In AD, impairment of mitochondrial function causes bioenergetic deficiency, intracellular calcium imbalance and oxidative stress, thereby aggravating the effect of Aβ and tau pathologies, leading to synaptic dysfunction, cognitive impairment and memory loss. Although there are reports suggesting intricate parallelism between mitochondrial dysfunction and AD pathologies such as Aβ aggregation and hyperphosphorylated tau accumulation, the factors that drive the pathogenesis of either are unclear. In addition, emerging evidence suggest that mitochondrial quality control (QC) mechanisms such as mitophagy are impaired in AD. As an important mitochondrial QC mechanism, mitophagy plays a critical role in maintaining neuronal health and function. Studies show that various proteins involved in mitophagy, mitochondrial dynamics, and mitochondrial biogenesis are affected in AD. Compromised mitophagy may also be attributed to impairment in autophagosome–lysosome fusion and defects in lysosomal acidification. Therapeutic interventions aiming to restore mitophagy functions can be used as a strategy for ameliorating AD pathogenesis. Recent evidence implicates the role of microglial activation via mitophagy induction in reducing amyloid plaque load. This review summarizes the current developments in the field of mitophagy and mitochondrial dysfunction in AD.

## Introduction

Alzheimer’s disease (AD) is one of the most debilitating age-induced neurodegenerative disorders affecting millions worldwide (Lane et al., 2018). It is characterized by extensive neuronal loss, synaptic dysfunction, mitochondrial damage, neuroinflammation, and accumulation of amyloid plaques and neurofibrillary tangles (NFTs) (Crews and Masliah, 2010; Butterfield et al., 2013; Kocahan and Dogan, 2017; Magalingam et al., 2018). Extensive research has gone into elucidating the mechanisms of the pathogenesis and development of this disease, with multiple dimensions and hypotheses being ascribed as causatives (Du et al., 2018). Amyloid beta (Aβ) fibrils and phosphorylated tau tangles are characteristic hallmarks of the disease, and these appear to spread through the cortex as the disease progresses (Davidson et al., 2018). In addition, several studies have highlighted the presence of damaged mitochondria and synaptic dysfunction as prior observations to the initiation of Aβ fibril or tau tangle formation (Cai and Tammineni, 2017; Guo et al., 2017; Tang et al., 2019). Therefore, the focus has now shifted to mitochondria as one of the central players in the pathogenesis of AD (Cadonic et al., 2016; Cai and Tammineni, 2017; Cardoso et al., 2017; Guo et al., 2017; Martin-Maestro et al., 2017; Swerdlow, 2018). Accumulation of damaged mitochondria might be a result of various insults including accrued Aβ oligomers or fibrils and phosphorylated tau. On the other hand, mitochondrial dysfunction due to mitochondrial DNA (mtDNA) damage or mutations, impairment in mitochondrial transport, or the like may lead to Aβ oligomeric or fibrillar formation and phosphorylated tau accumulation. Either way, a cause or consequence relationship draws attention to the fact that mitochondrial health and quality control (QC) is central to maintaining a healthy pool of neurons and that any damage to mitochondria might lead to neuronal loss, synaptic damage, and neurodegeneration (Lin and Beal, 2006; Johri and Beal, 2012; Hroudova et al., 2014; Bhat et al., 2015; Lane et al., 2015; Akbar et al., 2016; Kocahan and Dogan, 2017; Chen et al., 2019). Given the importance of maintaining healthy mitochondria, cells host a series of QC mechanisms to govern mitochondrial homeostasis. These major mitochondrial QC mechanisms include mitochondrial unfolded protein response (mtUPR), ubiquitin-proteasome system (UPS), mitochondrial-derived vesicle (MDV) degradation pathway, and mitophagy (a selective form of autophagy) (Ashrafi and Schwarz, 2013; Jovaisaite et al., 2014; Sugiura et al., 2014; Bragoszewski et al., 2017; Leites and Morais, 2018). Among these, mitophagy can selectively degrade the entire damaged mitochondria, and impairment of this pathway may lead to the development of AD (Kerr et al., 2017). Recent work has therefore focused on the contribution of mitochondrial dysfunction as a primary driving cause of AD.

## The Amyloid to Mitochondrial Cascade Hypothesis in the Pathogenesis and Progression of AD

The amyloid cascade hypothesis was first proposed by Hardy and Higgins (1992). According to this hypothesis, Aβ peptide deposition leads to the pathogenesis of AD, and the occurrence of NFTs, neuronal cell loss, vascular damage, and dementia is a direct result of this deposition (Hardy and Higgins, 1992). The observation of Aβ peptides in senile plaques of AD patients (Masters et al., 1985), along with the occurrence of the APP gene on chromosome 21, which causes Down’s syndrome (Hardy and Selkoe, 2002), initially invigorated the hypothesis. Although the amyloid cascade hypothesis has prevailed among others for decades, recent evidence points to the *de facto* role of Aβ peptide as a ‘seed,’ playing an important role in the development rather than the progression of the disease (Sowade and Jahn, 2017). The multifactorial nature of this disease also substantiates the fact that Aβ peptide might be required but not adequate for the development of AD (Dourlen et al., 2019). Further investigations in AD mouse models failed to establish a link between neuronal cell death and accumulation of senile plaques of Aβ fibrils (Bryan et al., 2009), possibly hinting that Aβ oligomers might be the key cytotoxic agents rather than the fibrillar form. In addition, later studies by Kim et al. (2007, 2013), using Aβ_42_ overexpressing BRI2-Aβ mice, showed that despite the presence of Aβ oligomers and Aβ amyloid fibrils, there was no impairment in cognitive function or degeneration of neurons. Furthermore, vast deposition of Aβ in the brains of elderly non-AD patients suggested that Aβ deposition might not be specific to AD (Price et al., 2009; Chetelat et al., 2013). The amyloid cascade hypothesis does not account for such observations, and thus several other alternatives have been proposed such as the tau hypothesis, cholinergic hypothesis, neuroinflammation hypothesis, and synaptic failure hypothesis (Lindwall and Cole, 1984; Okatsu et al., 2012; Kozlov et al., 2017; Kametani and Hasegawa, 2018). Although the previous notion of aggregate formation as the primary cause of the development and pathogenesis of AD still exists, focus is now shifting to the mitochondrial cascade hypothesis (summarized below). Mitochondrial dysfunction in the synapses has been commonly observed in AD, and studies are now emerging that evidently point to the role of dysfunctional mitochondria in the pathogenesis and progression of AD (Du et al., 2010; Cai and Tammineni, 2017; Guo et al., 2017; Pickett et al., 2018; Tang et al., 2019).

The mitochondrial cascade hypothesis was based on observations of glucose hypometabolism in the prodromal stage in AD patients, detected through fluorodeoxyglucose positron emission tomography (FDG PET) in early 1980s (Ferris et al., 1980; De Leon et al., 1983; Foster et al., 1983; Friedland et al., 1983). Theories have since emerged that point to the critical role of bioenergetic pathways in neuronal function. In fact, reduction in the activity of many enzymes related to mitochondrial bioenergetics, such as cytochrome c oxidase (COX), pyruvate dehydrogenase complex (PDHC), and α-ketoglutarate dehydrogenase complex (KGDHC), has been implicated in disease progression (Sorbi et al., 1983; Gibson et al., 1988; Parker et al., 1989). The mitochondrial cascade hypothesis was proposed by Swerdlow and Khan (2004) based on Parker’s initial proposal that mitochondrial function, determined by mtDNA inheritance, influences the risk of AD (Parker et al., 1989, 1990; Swerdlow and Khan, 2004). In summary, this hypothesis states that the age-related decline in mitochondrial function leads to various physiological changes in the neurons (Trifunovic et al., 2004; Navarro and Boveris, 2007). The cell tries to compensate and adapt to these changes, but upon reaching a threshold, such compensation is not possible, thus triggering symptoms characteristic of the disease. The clinical manifestation of AD is thus largely dependent on mitochondrial genetics and environmental influences. Based on these and many other lines of evidence, focus is now shifting towards mitochondrial dysfunction as the central player in the pathogenesis of AD (Swerdlow and Khan, 2004; Baloyannis, 2006; Lin and Beal, 2006; Eckert et al., 2011; Johri and Beal, 2012; Hroudova et al., 2014).

## Mitochondrial Dysfunction in AD—A Cause or an Effect?

Alzheimer’s disease is characterized by impairment in oxidative metabolism, free radical accumulation, reduction in mitochondrial metabolic enzyme levels, dysregulation of calcium homeostasis, and transcriptional and translational defects in mitochondria (Gibson et al., 2010). Aβ plaques and NFTs are the two hallmarks of AD. Aβ peptide is generated from the cleavage of amyloid precursor protein (APP) through the action of various secretases. APP is an integral transmembrane protein, and APP mRNAs are alternatively spliced to produce several isoforms of APP mRNAs, of which APP695 is the most abundant isoform in brain (Zheng and Koo, 2011). There are two pathways by which APP can be processed, the amyloidogenic and the non-amyloidogenic pathway, of which the amyloidogenic processing of APP predominantly leads to the increase in the Aβ_42_/Aβ_40_ ratio. The amyloidogenic processing of APP begins with the cleavage of the precursor protein by β-secretases followed by γ-secretases, both of which are enriched in the trans-Golgi network and endosomal compartment, leading to the generation of secreted APP (sAPPβ), C-terminal fragment 99 (CTF 99), and Aβ peptide fragments. The fragments thus generated may vary in length from 38 to 43 amino acids, some of which are more pathogenic than others (Chow et al., 2010).

Another hallmark of AD is NFTs comprised primarily of tau, a microtubule-associated protein (MAP) enriched in the axonal regions of neurons. Hyperphosphorylated forms of tau are responsible for their aggregation into paired helical filaments (PHFs) and subsequent accumulation into tangles, leading to the degeneration of neurons (Lindwall and Cole, 1984; Okatsu et al., 2012; Kozlov et al., 2017; Kametani and Hasegawa, 2018). The MAP tau protein exists in six isomeric forms ranging from 352 to 441 amino acids due to alternative splicing of exons 2, 3, and 10 (Kametani and Hasegawa, 2018). Exon 10 harbors the microtubule-binding domain. In normal physiological form, tau binds to tubulin and stabilizes the microtubules (Iqbal et al., 2010). Since tau is a phosphoprotein, its activity is largely dependent on its phosphorylation status (Iqbal et al., 2010). Mutations in tau protein lead to its hyperphosphorylation and mislocalization to the cell body or dendrites, where it forms NFTs or straight filaments (SFs), respectively (Zempel and Mandelkow, 2014). Impairment of the synaptic vesicle cycle (Zhou et al., 2017), mitochondrial dysfunction (DuBoff et al., 2012), and cytoskeletal disintegration (Fulga et al., 2007) also occur as a result of NFT or SF accumulation.

While there are reports suggesting that dysfunctional mitochondria drive Aβ and hyperphosphorylated tau pathology (Swerdlow, 2018; Albensi, 2019), contradictory studies also hint at Aβ and pathogenic tau-driven mitochondrial dysfunction (Wang et al., 2007; Mossmann et al., 2014; Todd et al., 2014). Whether mitochondrial dysfunction leads to AD or the pathologies underlying the disease subsequently cause mitochondrial dysfunction is a well-debated topic. Additionally, the possibility of a feedback loop between the two cannot be ruled out. Some of the cause and consequence relationships between mitochondrial dysfunction and pathological development of AD are summarized below, with a special focus on Aβ plaques and hyperphosphorylated tau.

### Evidence for Mitochondrial Dysfunction Leading to Amyloid Beta and Pathological Tau Accumulation

Evidence for mitochondrial dysfunction as the causative agent in Aβ and tau-aided AD development has been reported using various cell lines as well as mouse models. A study by Gabuzda et al. (1994) showed that inhibiting the energy metabolism in COS cell line using sodium azide and carbonyl cyanide m-chlorophenylhydrazone shifted the processing of APP toward Aβ production. Similar studies have indicated the importance of cellular bioenergetics, the dysregulation of which can potentially drive the defective processing of APP and more Aβ production (Webster et al., 1998; Gasparini et al., 1999).

Cybrid (cytoplasmic hybrid) cell lines have been used consistently in support of the primary mitochondrial cascade in AD. Cybrid cells were initially created to address the question of whether reduced COX activity in platelets derived from AD patients could be attributed to mitochondrial DNA (mtDNA). Cybrid cell lines were made from SH-SY5Y or NT2 cell lines by removing their endogenous mtDNA and fusing them with platelet cells from AD or age-matched control patients in the presence of a detergent. The mitochondrial DNA-depleted SH-SY5Y cells were termed p^0^ cells. The cybrid cells were then selected for mitochondria derived from platelets and for nuclear DNA derived from p^0^ cells. These cybrid cell lines derived from AD patients and those derived from control patients differed in their mtDNA content alone, and thus any difference in the physiological status of the cells between AD or control cybrids could be attributed to their mtDNA (Sheehan et al., 1997; Scheffler et al., 2012). Such studies have also been carried out using transgenic APP mouse models that differed in their mitochondrial contents (Khan et al., 2000; Onyango et al., 2010; Scheffler et al., 2012). In all these studies, the cybrids that contained mitochondria from AD patients showed a significant increase in the levels of Aβ as compared to age-matched control cybrids. Also, the mean COX activity between groups of AD and age-matched controls differed in that the mean was lower in the AD group than in the control group. While some may argue that this difference may be driven by the transfer of APP or Aβ, the cybrid data suggests otherwise, hinting at the role of mtDNA in reducing the mitochondrial platelet COX activity (Sheehan et al., 1997; Swerdlow and Khan, 2004).

Reactive oxygen species (ROS) are generated as a by-product of the electron transport chain in mitochondria (Murphy, 2009). ROS serve as signaling molecules, but levels beyond their physiological threshold can induce mitochondrial dysfunction and can eventually lead to ROS-associated cellular damage (Zorov et al., 2014). Experimental evidence of inducing mitochondrial damage subsequently leading to Aβ buildup inside cells suggested a possible role of mitochondrial dysfunction in triggering AD. A study found that induction of mitochondrial damage by rotenone and antimycin A, which act on complexes I and III, increased ROS levels. Interestingly, the treated cells also showed significant levels of soluble Aβ (Leuner et al., 2012).

Likewise, studies using two *in vivo* mouse models also demonstrated the appearance of AD-like symptoms upon perturbation of mitochondrial function. Ndufs4-null (KO) mice, in which mitochondrial complex I was absent, showed elevated levels of Aβ_40_ as compared to age-matched control mice, with progressive development of ataxia and death at week 7. A similar increase in Aβ_40_ levels was seen in a Thy-1 APP mouse model when treated with rotenone for 3 days (Leuner et al., 2012). However, a reduction in Aβ levels was observed in an AD mouse model crossed with a complex IV-null model, with a concomitant decrease in ROS levels, thus suggesting that mitochondria-derived ROS is the key to more Aβ production (Fukui et al., 2007).

In an *in vivo* mouse model, D257A;APP/Ld, which carries a mutation with an abolished proofreading function of mitochondrial DNA polymerase γ in an AD background, it was observed that the levels of Aβ_42_ and the plaque density were increased, leading to age-related phenotypes and death within a year after birth (Kukreja et al., 2014). The mitochondrial DNA of this transgenic AD mice harbored extensive mutations leading to mitochondrial dysfunction, thus linking the pathogenesis of AD primarily to defective mitochondria and dysfunctional bioenergetics (Kukreja et al., 2014). These and other such studies (Sims et al., 1985, 1987; Gibson et al., 1988) have led to the proposition of defective mitochondria being the causative agent of the development and pathogenesis of AD.

In parallel, studies have also shown how mitochondrial dysfunction can lead to tau hyperphosphorylation and accumulation into tangles. The phosphorylation status of the microtubule-associated domain of MAPT (microtubule-associated protein tau) determines its binding capability with the microtubule (Mazanetz and Fischer, 2007). Hyperphosphorylation of serine, threonine, and tyrosine residues may lead to destabilization and dissociation of MAPT from microtubules and cause the equilibrium to shift towards aggregate formation (Neddens et al., 2018). Mitochondrial dysfunction leading to oxidative stress can be one of the major causes of tau hyperphosphorylation. This was attributed to the inhibition of glutathione synthesis using buthionine sulfoximine (BSO), which caused a significant increase in the activity of kinases like JNK and p38 and a decrease in the activity of phosphatases such as PP2A, thus leading to hyperphosphorylation of tau in M17 neuroblastoma cells in a time-dependent manner (Su et al., 2010). In another *Drosophila* model of human neurodegeneration, expression of mutant human tau (tau^R406W^) under a pan-neuronal driver, elav-GAL4, caused extensive tau hyperphosphorylation via p38-MAPK activation (Dias-Santagata et al., 2007). The role of kinases and phosphatases, namely, GSK3β and PP2A, has also been implicated in ROS production and tau hyperphosphorylation (Abou-Sleiman et al., 2006; King et al., 2008; Feng et al., 2013), thus substantiating the primary mitochondrial cascade hypothesis in the development of AD.

### Evidence for Mitochondrial Dysfunction Due to Amyloid Beta and Pathological Tau Accumulation

**FIGURE 1:**
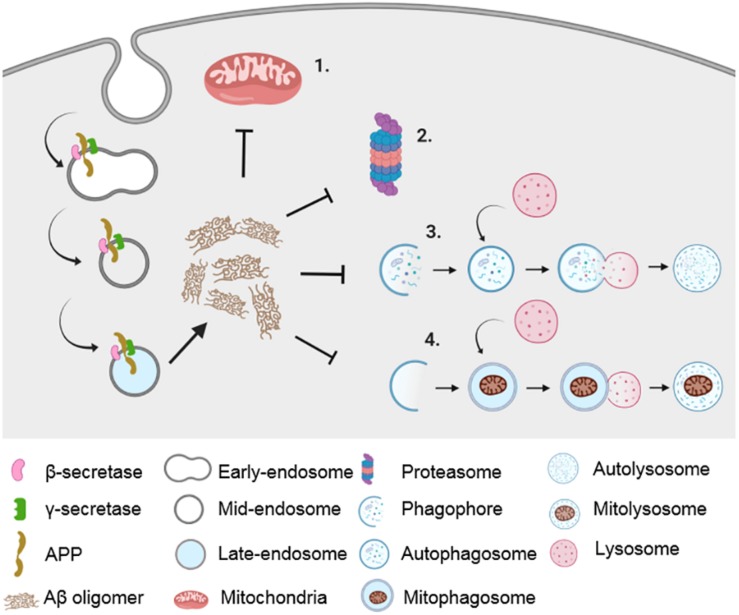
Impairment in proteostatic machineries and mitochondrial dysfunction in AD. Amyloidogenic processing of APP in the endocytic pathway results in the generation of Aβ peptide, which causes impairment in mitochondrial function (1) as well as in various proteostatic pathways such as UPS (2), autophagy (3), and mitophagy (4).

Contrary to the previous evidence highlighting the contribution of mitochondrial damage to the progression of AD, a plethora of studies showed the role of Aβ and pathogenic tau in abrogating mitochondrial function. Studies have shown that pathogenic Aβ peptide generation causes dysfunction in mitochondrial function as well as in various proteostatic pathways (Figure 1). The first evidence of Aβ-induced mitochondrial dysfunction was provided by Cardoso and colleagues, where external addition of Aβ to a medium containing NT2 cells or NT2 p^0^ cells (with mtDNA removed) showed differential toxicity to Aβ. Since NT2 p^0^ cells lack respiratory enzymes, it was proposed that Aβ-induced mitochondrial dysfunction was mediated through the respiratory pathway (Cardoso et al., 2001).

An Aβ-induced defect in mitochondrial function was shown via a reduction in respiratory functions and electron transport chain enzyme activities (Casley et al., 2002). Subsequently, several studies, using both cultured cells and *in vivo* models, as highlighted below, have demonstrated the detrimental effects of Aβ and pathogenic tau on mitochondria. In addition, AD-patient brain autopsies showed the presence of Aβ in mitochondria (Manczak et al., 2011). Aβ has been shown to cause mitochondrial dysfunction in the following ways: through APP-mitochondria interactions, through direct interaction with mitochondrial enzymes, through direct insertion into mitochondrial membrane pore, through a calcium cascade, or through inhibiting the fission–fusion dynamics of mitochondria. These are further discussed below.

The Aβ precursor protein APP contains a binding motif for TOM40 (translocase of the outer mitochondrial membrane 40 kDa) that inhibits its activity and impairs normal functioning of the mitochondria (Anandatheerthavarada et al., 2003; Anandatheerthavarada and Devi, 2007). APP binding to TOM40 also inhibits the entry of COX subunits IV and Vb, thus reducing the activity of COX and leading to an increase in ROS production (Manczak and Reddy, 2012a). In a study conducted by Lustbader et al. (2004), it was found that Aβ could bind to a mitochondrial dehydrogenase enzyme, ABAD (Aβ binding to alcohol dehydrogenase), preventing NAD binding. Drugs interfering with Aβ-ABAD interaction were effective in improving memory and cognitive functions in AD transgenic mice (Lustbader et al., 2004). In another study, Aβ was found to insert into the mitochondrial transition pore component cyclophilin D (CyPD) and impair mitochondrial function. This was attenuated in CyPD knockout mice, improving their cognitive functions (Du et al., 2008). Through a different mechanism, Sanz-Blasco et al. (2008) showed that exogenously added oligomeric Aβ led to an influx of extracellular calcium and an overloading of calcium onto mitochondria, leading to mitochondrial dysfunction characterized by Δψ subsidence, opening of mitochondrial permeability transition pore (mtPTP) channels, and release of cytochrome c, eventually causing cell death by apoptosis.

**FIGURE 2:**
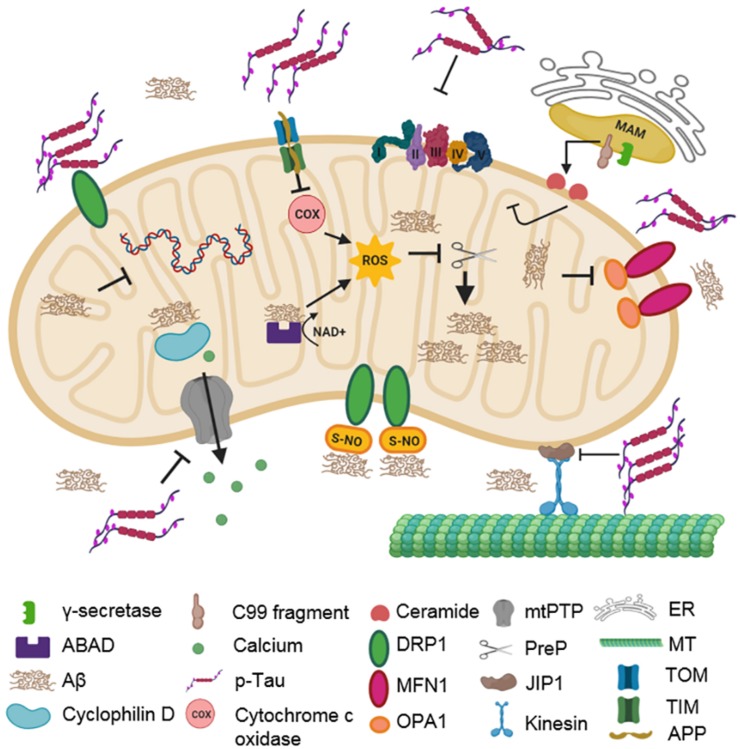
Impact of AD-associated protein aggregates on mitochondrial integrity. Aβ oligomers and hyperphosphorylated tau cause mitochondrial dysfunction. Aβ oligomers inhibit COX, ABAD, and PreP functions. They also impair fission–fusion dynamics by changing the levels of DLP1, OPA1, and MFN1. CyPD function is also inhibited, disturbing mitochondrial permeability. Hyperphosphorylated tau compromises mitochondrial transport, dynamics, and permeability. It stalls mitochondrial transport along microtubules by inhibiting JIP1. It also interacts with DLP1, OPA1, MFN1, and MFN2, thereby affecting mitochondrial dynamics. Additionally, it can interact with VDAC1, which affects the opening and closing of mtPTP, thereby impairing membrane permeability. C99 fragments in MAM can generate ceramides due to increased sphingomyelinase activity.

Elevated levels of Aβ can also influence the fission–fusion dynamics of mitochondria. Aβ has been shown to enhance S-nitrosylation of dynamin-like protein-1 (DLP1/DRP1), leading to increased mitochondrial fission and subsequent loss from dendrites and axonal regions (Cho et al., 2009). Also, there is increased mitochondrial fission upon Aβ expression in cells and in *in vivo* transgenic mouse models (Wang et al., 2009). However, a study by DuBoff et al. (2012) found that mutated human tau caused actin stabilization and prevented DRP1 localization to mitochondria, causing mitochondrial fusion and neurotoxicity. Other studies have shown that expression of APP695 in M17 neuroblastoma cell line showed an increase in the level of mitochondrial fission 1 protein (FIS1), a fission protein, and decreased levels of mitofusin 1 (MFN1) and OPA1, proteins involved in fusion. The mitochondria in these cells were reportedly fragmented, and the dynamics were slower (Wang et al., 2008). In yet another study, it was shown that a particular mitochondrial protease called presequence protease (PreP), known to degrade Aβ, was inactivated through Aβ-induced oxidative stress mechanisms, thereby further increasing Aβ concentration in mitochondrial matrix (Alikhani et al., 2009). This evidence points to the possibility of a secondary mitochondrial cascade wherein pathogenic Aβ accumulation leads to mitochondrial dysfunction, thereby aggravating the disease phenotype (Figures 1, 2).

An interesting hypothesis that has recently emerged is that of the mitochondria-associated ER membrane (MAM) contributing to AD pathology (Area-Gomez et al., 2018). MAM is a subdomain of the ER that serves as a contact site between mitochondria and ER and is especially enriched in cholesterol and sphingomyelin, thus imparting the characteristic features of lipid rafts (Hayashi and Su, 2010). Surprisingly enough, it was found that presenilin and γ-secretase, as well as Aβ peptide-generation, was enriched in MAMs (Newman et al., 2014; Schreiner et al., 2015; Del Prete et al., 2017). Since MAM serves important functions in calcium transport, synthesis of phospholipids, mitochondrial fission–fusion dynamics, division of mtDNA, and cholesterol esterification (Hayashi et al., 2009), several studies have investigated the role of mitochondrial dysfunction in AD mediated through Aβ via MAM.

It was subsequently found that the C99 fragment responsible for the generation of Aβ_42_ was present not only in endosomes, as expected, but also in MAMs. Since γ-secretase is also found in MAMs, the amyloidogenic processing of C99 to Aβ_42_ has been proposed to take place in MAMs (Schreiner et al., 2015; Pera et al., 2017). Furthermore, the accumulation of the C99 fragment led to increased sphingomyelinase (SMase) activity in MAMs (Pera et al., 2017), thereby altering its structure and function, leading to the generation of ceramides, which can cause mitochondrial dysfunction via apoptosis or inhibition of mitochondrial respiration (Haimovitz-Friedman et al., 1997; Yu et al., 2007). A simultaneous increase in the SMase activity in SH-SY5Y cells upon inhibition of γ-secretase was seen in MAM domains, with a concomitant increase in C99 levels (Pera et al., 2017). Supporting this result, the inhibition of beta-site APP-cleaving enzyme 1 (BACE1) activity (which reduces C99-formation) resulted in an attenuation of SMase activity (Pera et al., 2017). Area-Gomez et al. (2012) thus proposed a mechanism whereby mitochondrial dysfunction lies downstream of C99 accumulation in the MAM. Although this hypothesis does not account for a direct effect of Aβ_42_ on mitochondrial dysfunction, the observed accumulation of C99 fragments on mitochondrial MAMs and increased ER-mitochondrial connectivity (Area-Gomez et al., 2012) suggest an early role of mitochondrial dysfunction in AD. Hyperphosphorylated tau can similarly impair mitochondrial functioning in three major ways, namely, (i) by shifting the equilibrium of mitochondrial fission–fusion towards increased fission, (ii) by impairing transport of mitochondria, and (iii) by causing dysfunction in oxidative phosphorylation and increasing ROS production (Figure 2). Increased fission in mitochondria due to the presence of hyperphosphorylated tau has been attributed to the atypical interaction between tau and DRP1, observed in brain tissues of APP, APP/PS1, and 3xTg-AD mice and AD patients (Manczak and Reddy, 2012a). Moreover, reducing DRP1 levels protected against mutated tau-induced synaptic impairment (Kandimalla et al., 2016). Li et al. (2016) have shown that expressing full-length wild-type tau caused disruption in mitochondrial function via increased fusion in HEK293 and rat primary hippocampal neurons mediated by an increase in the level of fusion proteins such as OPA1, MFN1, and MFN2, thereby causing cellular damage and degeneration. It has also been proposed that mitochondrial dysfunction due to tau pathology can be divided into two distinct phases: an early phase wherein tau mediates a protective function by promoting mitochondrial fusion and a late phase where increased mitochondrial fission leads to degeneration of neurons (Wang et al., 2014).

In a squid model of AD, it was found that the filamentous form of hyperphosphorylated tau inhibited the transport of mitochondria along axons through activation of glycogen synthase kinase (GSK3) and protein phosphatase 1 (PP1) (Kanaan et al., 2011). Also, mitochondrial transport in cortical neurons was influenced by overexpression of GSK3β and the p25 activator of cyclin dependent kinase 5 (CDK5) (Morel et al., 2010). Mutated tau with hyperphosphorylation in AT8 sites also showed impaired mitochondrial transport through inhibition of c-Jun NH2-terminal kinase (JNK) interacting protein 1 (JIP1) (Ittner et al., 2009). It has also been shown that mutated tau can interact with voltage-dependent anion-selective channel 1 (VDAC1) and influence the opening and closing of mtPTP, and disrupt mitochondrial membrane potential, thereby causing dysfunction (Manczak and Reddy, 2012a). Several studies have also indicated increased ROS formation and dysfunctional oxidative phosphorylation systems in the presence of mutated tau (Rhein et al., 2009; Eckert et al., 2011; Schulz et al., 2012; Mondragon-Rodriguez et al., 2013; Alavi Naini and Soussi-Yanicostas, 2015).

In view of the above evidence, mitochondrial dysfunction and Aβ/pathological tau accumulation seemingly appear physically and temporally connected. Since mitochondrial dysfunction leads to the accumulation of damaged mitochondria, various QC mechanisms are imperative for maintaining healthy neuronal populations; these are summarized below.

## Role of Mitochondrial Quality Control Mechanisms in Maintaining Cellular Homeostasis

Since mitochondrial function and integrity are critical parameters for maintaining cellular homeostasis, cells have evolved several mitochondrial QC mechanisms to maintain a healthy pool of functional mitochondria. Apart from functioning independently in response to specific cues, there exists a crosstalk between these mitochondrial QC pathways. The activation of specific mitochondrial QC mechanisms is dependent on the extent of mitochondrial damage occurring at both molecular and organellar levels. This makes mitochondrial QC mechanisms an interdependent hierarchical system that monitors mitochondrial integrity, thereby ensuring the survival of cells (Rugarli and Langer, 2012). Mitochondrial damage can be induced by various factors like ROS, abnormal protein aggregates (Aβ, tau), mutations in genes encoded by the mitochondrial and nuclear genome, and exposure to toxic drugs (Angelini et al., 2009; Tuppen et al., 2010; Fischer et al., 2012; Manczak and Reddy, 2012a, b; Vuda and Kamath, 2016). The various mitochondrial QC mechanisms that are initiated depending on the degree of mitochondrial damage caused by these insults are summarized below.

### mtUPR, Ubiquitin-Proteasome System, MDVs, and Mitophagy

The first line of defense in response to mitochondrial protein damage involves the activation of various mitochondrial resident chaperones and proteases, which help in maintaining mitochondrial proteostasis. The mitochondrial chaperones heat shock protein 22 (HSP 22), HSP 60, and HSP 70 help in the refolding of the misfolded proteins to their native three-dimensional conformation, thereby maintaining protein functionality (Baker et al., 2011). In contrast, irreversibly damaged proteins are degraded by a set of mitochondrial resident proteases, especially the Lon proteases and Clp proteases. These ATP-dependent proteases recognize the exposed hydrophobic regions in the denatured proteins and degrade them after unfolding (Sauer and Baker, 2011). Mutations in genes encoding these proteases and chaperones lead to neurological disease phenotypes like hereditary spastic paraplegia, spastic ataxia neuropathy syndrome, and spinocerebellar ataxia (Atorino et al., 2003; Levytskyy et al., 2016). However, if the level of misfolded and damaged proteins rises above a threshold, it activates a stress response called mtUPR. The activation of mtUPR results in elevated expression of nuclear genes encoding mitochondrial chaperones and proteases, thereby reducing the concentration of the damaged proteins (Zhao et al., 2002; Haynes and Ron, 2010).

Secondly, damaged mitochondrial proteins can also be degraded with the help of the cytoplasmic 26S proteasome system. Here, the damaged outer mitochondrial membrane (OMM) proteins are retro-translocated from the membrane with the help of p97, an AAA+ ATPase, and are degraded by the cytoplasmic proteasome system (Tanaka et al., 2010; Xu et al., 2011).

The next level of mitochondrial QC becomes activated when there is localized or severe damage to mitochondria. The two mechanisms that function in response to these forms of damage are MDVs and mitophagy, constituting the third and fourth levels of mitochondrial QC. MDVs are 70–150-nm-sized vesicles that bud off from the mitochondria, selectively incorporating large assemblies of damaged proteins and lipids from the OMM, inner mitochondrial membrane (IMM), and matrix (Sugiura et al., 2014). They are usually formed when there is local accumulation of damaged proteins resulting in blockage of mitochondrial import channels. The MDVs containing damaged mitochondrial proteins are targeted to either lysosomes, late endosomes, multivesicular bodies, peroxisomes or they undergo exocytosis (Sugiura et al., 2014). The field of MDVs is relatively underexplored.

Mitophagy, the next organellar level of mitochondrial QC, differs from the above-mentioned pathways in that it can target an entire damaged mitochondrion for degradation. It is a selective autophagy pathway that recognizes damaged mitochondria and sequesters them into autophagosomes, thereby forming mitophagosomes that eventually fuse with lysosomes and are degraded (Palikaras et al., 2018). Different mitophagy pathways become activated upon specific cues, and a plethora of proteins are involved in the execution of these pathways. The molecular details of mitophagy pathways are discussed briefly below.

#### PINK1-Parkin Pathway

The PINK1-Parkin pathway is one of the best-characterized stress-induced mitophagy pathways. PINK1 is a serine/threonine kinase that functions as a sensor for mitochondrial health (Unoki and Nakamura, 2001). In healthy mitochondria, PINK1 gets imported into mitochondria through TOM (Translocase of Outer Membrane) and TIM (Translocase of Inner Membrane) complexes, respectively. Once PINK1 has been imported into the IMM, it is processed by two proteases, MPP (matrix processing peptidase) and PARL (rhomboid protease presenilin-associated rhomboid-like), thereby making it a substrate for N-end rule degradation by the cytosolic UPS (Jin et al., 2010; Deas et al., 2011; Greene et al., 2012; Yamano and Youle, 2013). When mitochondria are damaged, PINK1 import is blocked, and it accumulates on the OMM, which spares it from proteolytic processing (Jin et al., 2010; Narendra et al., 2010; Lazarou et al., 2012; Jin and Youle, 2013). The accumulated PINK1 autophosphorylates itself, making its kinase domain-active (Okatsu et al., 2012). Upon activation, PINK1 phosphorylates its substrate proteins MIRO (mitochondrial rho GTPase), MFN1, and ubiquitin, which promotes the recruitment of parkin, a cytosolic E3 ubiquitin ligase, onto the damaged mitochondria (Chen and Dorn, 2013; Kane et al., 2014; Shlevkov et al., 2016). The binding of parkin to phosphorylated ubiquitin (pS65Ub), followed by PINK1 phosphorylation at serine 65, promotes parkin activation, which then ubiquitinates its downstream targets (Zhang et al., 2012; Kane et al., 2014; Koyano et al., 2014; Nguyen et al., 2016). Ubiquitination of OMM proteins by parkin provides PINK1 with more substrates for phosphorylation, which further promotes enhanced recruitment of parkin and its activation, forming a positive feedback loop (Ordureau et al., 2014). K63 ubiquitination by parkin recruits autophagy adaptor proteins like optineurin (OPTN), nuclear dot protein 52 (NDP52), Tax1-binding protein 1 (TAX1BP1), sequestosome-1 (SQSTM1)/p62, and neighbor of BRCA1 gene 1 (NBR1) to damaged mitochondria (Sarraf et al., 2013; Lazarou et al., 2015; Nguyen et al., 2016). These adaptor proteins mediate the recruitment of autophagy machinery to the damaged mitochondria and interact with the autophagosomal proteins LC3 (microtubule-associated protein 1A/1B-light chain 3) or GABARAP (gamma-aminobutyric acid receptor-associated protein), eventually forming a mitophagosome (Nguyen et al., 2016). The mitophagosomes subsequently fuse with lysosomes, where the damaged mitochondria are degraded.

#### Receptor-Mediated Mitophagy

In addition to the PINK1-Parkin pathway, certain mitophagy receptor proteins, mostly integral mitochondrial proteins, can interact with autophagy machinery to mediate mitophagy. These include the OMM proteins FUNDC1 (FUN14 domain containing 1), BNIP3 (BCL2 interacting protein 3), NIX (Nip3-like protein X), Bcl2L13 (Bcl2-like protein 13), and FKBP8/FKBP38 (FK506−binding protein 8), and the IMM phospholipid cardiolipin (Rodger et al., 2018). These proteins promote mitophagy in a PINK1-Parkin independent manner in response to various cellular stimuli ranging from stress signals like hypoxia to developmental signals during differentiation of erythrocytes, retinal ganglion cells (RGCs), and cardiomyocytes, and also during reprogramming of somatic cells to iPSCs (Schweers et al., 2007; Liu et al., 2012; Gong et al., 2015; Esteban-Martínez et al., 2017; Xiang et al., 2017). Despite having an efficient mitochondrial QC system functioning in cells during various disease conditions especially in neurodegenerative diseases, these pathways usually become affected or impaired, which is discussed in detail in the following section.

## Impairment of Mitochondrial QC in AD

Neurons showing abnormal accumulation of damaged mitochondria and autophagic vacuoles in soma, axons, synapses, and degenerating neurites is one of the prominent phenotypes seen in AD (Nixon et al., 2005; Baloyannis, 2006; Trushina et al., 2012; Cai and Tammineni, 2016). This indicates that mitochondrial QC could be compromised in AD. Mounting evidence suggests impaired mitophagy as one of the contributing factors in AD pathogenesis (Kerr et al., 2017). Therefore, the functional status of mitochondrial QC in the context of AD is discussed below.

mtUPR, the mitochondrial QC that helps maintain mitochondrial proteostasis, is shown to be chronically activated in sporadic and familial AD patient brains. Sporadic AD patients showed ∼40–60% upregulation of mtUPR genes, while familial AD patients showed ∼70–90% upregulation (Beck et al., 2016). Chronic activation of this pathway could be a compensatory neuroprotective mechanism against the aberrant accumulation of misfolded and damaged mitochondrial proteins as well as the toxic protein aggregates during AD pathogenesis (Beck et al., 2016). However, sustained activation of this pathway in a chronic state could possibly shift its protective role to a deleterious one. Therefore, further investigations are required to understand the role of this sustained chronic activation of mtUPR in AD.

The activity of the cytosolic 26S proteasome system, which can selectively degrade damaged OMM proteins, was shown to be impeded in AD (Bonet-Costa et al., 2016). A mutant form of ubiquitin Ub^+1^ that is selectively detected in AD patient brains inhibits the degradation of polyubiquitinated substrates by 26S proteasome (Lam et al., 2000). This inhibition of 26S proteasome in AD can adversely affect both mitochondrial and cellular proteostasis, further contributing to the accumulation of abnormal protein aggregates, thus exacerbating the AD pathogenesis.

Recent reports suggest an impaired mitophagy pathway as a potential contributing factor in AD. Studies investigating the role of parkin-dependent mitophagy in AD revealed that, in mutant hAPPTg neurons and AD patient brains, there is increased recruitment of parkin, LC3, and p62 to damaged mitochondria. This suggests the induction of mitophagy in the early stages of disease progression (Ye et al., 2015). However, mutant hAPPTg neurons showed aberrant accumulation of mitophagosomes and increased retention of damaged mitochondria in enlarged and clustered LAMP1 (lysosomal-associated membrane protein 1)-positive vesicles. This indicates that despite the induction of mitophagy in early stages, the degradation of cargo is not very efficient, possibly due to compromised lysosomal function (Orr and Oddo, 2013; Kerr et al., 2017). Studies showed that familial AD PS1 (presenilin 1) mutations increased lysosomal alkalization and decreased lysosomal hydrolytic activity, thereby implicating compromised lysosomal function as a contributing factor in AD (Coffey et al., 2014). Additionally, with disease progression, there is significant reduction in cytosolic parkin levels in AD patient brain samples, which is indicative of inefficient mitophagy (Ye et al., 2015; Cai and Tammineni, 2016). Therefore, this evidence suggests that mitophagy impairment in AD could be a combined effect of compromised lysosomal function and decreased mitophagy proteins.

Parkin-dependent mitophagy is also affected in tau-mediated AD pathogenesis. A recent study showed that the expression of both human wild-type (htau) and mutant tau (hP301L) in neuroblastoma cells reduced parkin translocation to damaged mitochondria. The reduction in parkin translocation was significantly higher in cells expressing mutant tau (Cummins et al., 2019). This reduction in parkin translocation is due to the aberrant interaction of the projection domain of tau with parkin, thereby sequestering it in the cytosol. This was also shown *in vivo* in the *C. elegans* nervous system, wherein htau expression reduced mitophagy while the mutant tau completely inhibited mitophagy (Cummins et al., 2019). Therefore, aberrant interaction of tau with parkin can impair mitophagy, contributing to AD pathology, in addition to the occurrence of tau-mediated mitochondrial dysfunction.

**FIGURE 3:**
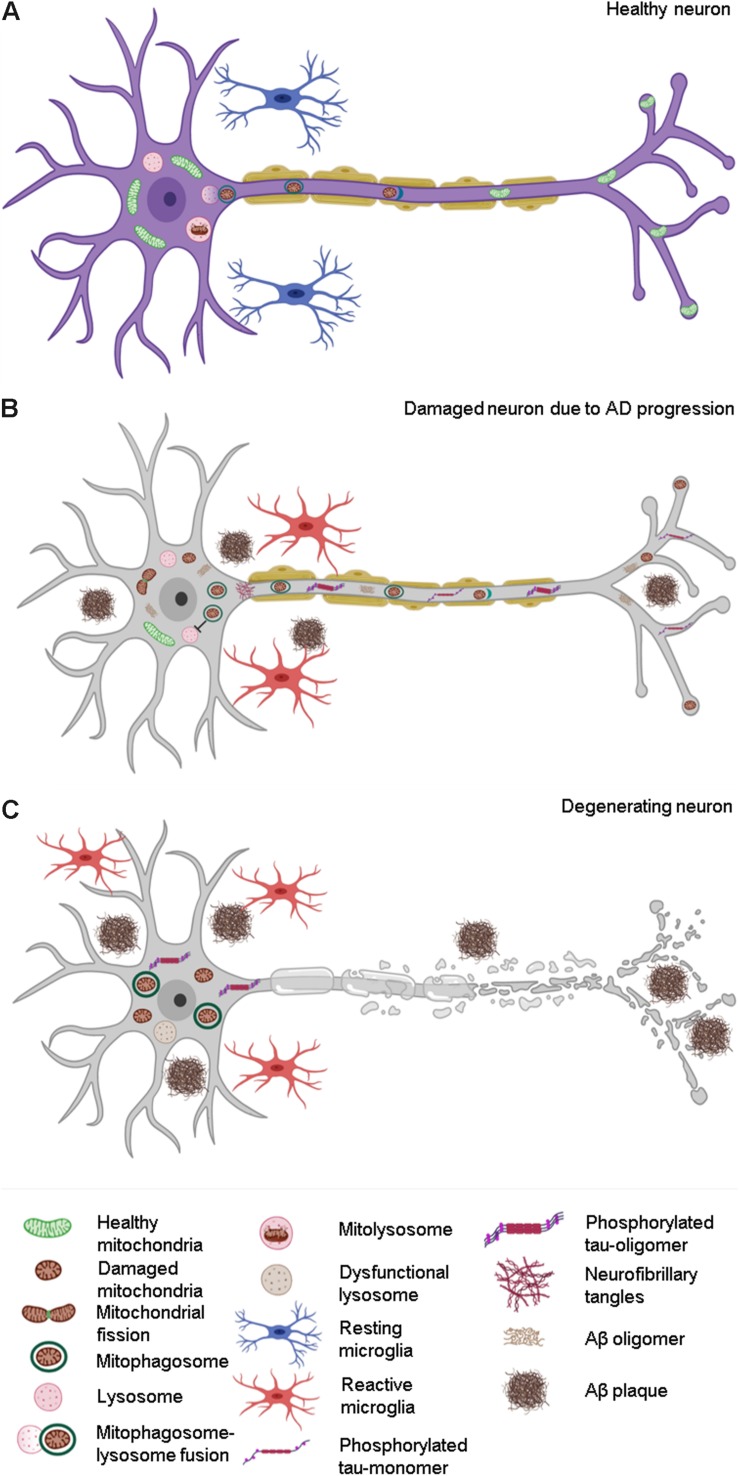
Effect of compromised mitophagy on neurons and microglia during AD progression. **(A)** Neurons and microglia maintain a healthy pool of mitochondria with the help of effective quality control (QC) mechanisms such as mitophagy. **(B)** Compromised mitophagy of AD-affected neurons leads to accumulation of damaged mitochondria and mitophagosomes. Mitophagy is also impaired in activated microglia, which contributes to their reduced phagocytic efficiency during AD pathogenesis. **(C)** Degeneration of neurons during the late stages of AD due to increased Aβ and tau pathology.

A recent study by Fang et al. (2019), showed that the levels of mitophagy-related proteins Bcl2L13, PINK1, and BNIP3L/NIX were reduced and mitophagy initiation proteins such as phospho-ULK1 (Ser555), and phospho-TBK1 (Ser172) were inactivated in AD patient samples. This reduction in mitophagy-related proteins was also shown in iPSC-derived cortical neuronal cultures generated from familial (APP/V717L) and sporadic AD (apolipoprotein E4 (APOE4)/E4) patients. Additionally, in iPSC-derived cortical neurons, the levels of other mitophagy-related proteins like FUNDC1, Bcl2L13, AMBRA1 (BECN1-regulated autophagy protein 1), and MUL1 (mitochondrial ubiquitin ligase activator of NFKB-1) were also shown to be decreased, further confirming the impairment of mitophagy in AD. Another study by Martin-Maestro et al. (2019) also reported the impairment of the mitophagy pathway in APP- and tau-overexpression models. This study showed a reduction in PINK1 and parkin translocation to damaged mitochondria in APP- and tau-overexpression models, suggesting the role of compromised mitophagy in the accumulation of damaged mitochondria in AD models. Apart from neurons, mitophagy in microglia was also shown to be reduced by ∼60% in the hippocampus of AD mouse models with a concomitant increase in damaged mitochondria (Fang et al., 2019). As microglia play an important role in high energy requiring functions like phagocytosis, an increase in damaged mitochondria due to compromised mitophagy might lead to subsequent energy deficiency, making the microglia-associated functions less efficient (Figure 3). Since a reduction in levels of mitophagy-related proteins and a concomitant decrease in mitophagy contribute to AD pathogenesis, gene therapy-mediated overexpression of mitophagy-related proteins could potentially increase mitophagy flux and thereby have beneficial effects (Khandelwal et al., 2011; Du et al., 2017).

Besides the proteins involved in mitophagy, mitochondrial dynamics and biogenesis-related proteins have also been shown to be linked with AD pathogenesis. Reports show cases of excessive mitochondrial fission and decreased mitochondrial fusion in AD (Cai and Tammineni, 2016). Mitochondrial fission proteins like DRP1, mitochondrial fission factor (MFF), mitochondrial dynamics protein (MiD51), FIS1, and MiD49 were shown to be upregulated in AD, causing excessive fragmentation of the mitochondrial network (Oliver and Reddy, 2019). It was also shown that elevated levels of S-nitrosylated DRP1 and aberrant interaction of DRP1 with Aβ and phosphorylated tau further lead to increased mitochondrial fission (Cho et al., 2009; Manczak and Reddy, 2012a). In addition to the upregulation of fission proteins, mitochondrial fusion proteins like MFN1, MFN2, and OPA1 are shown to be downregulated in AD (Oliver and Reddy, 2019). Therefore, excessive fission combined with the downregulation of mitochondrial fusion can lead to bioenergetic inefficiency, which can contribute to neuronal dysfunction in AD.

The expressions of genes associated with mitochondrial biogenesis like *PGC-1*α, *NRF2*, and *TFAM* are shown to be reduced in postmortem brain tissue samples obtained from AD patients (Rice et al., 2014; Kerr et al., 2017). Sirtuins, an evolutionarily conserved family of NAD^+^-dependent deacetylases that regulate multiple cellular pathways including mitochondrial biogenesis and mitophagy, are also known to be affected in AD. SIRT1 is a nuclear sirtuin that plays a role in the upregulation of PGC-1α and the activation of autophagy/mitophagy genes such as *ATG7, ATG6, LC3*, and *NIX/BNIP3L* (Fang et al., 2016; Kerr et al., 2017). Studies have shown the levels of SIRT1 to be significantly reduced in the parietal cortex of AD patient brain samples. Further analysis suggests a negative correlation of SIRT1 mRNA and protein levels with tau accumulation and disease progression (Julien et al., 2009). Levels of SIRT3, a mitochondrial sirtuin that has a role in p62 clustering onto ubiquitinated mitochondria and autolysosome formation, were reduced in AD (Tseng et al., 2013; Yang et al., 2015). Overall, these findings suggest that impairment in mitochondrial QC combined with abnormal mitochondrial dynamics and biogenesis can contribute to AD pathogenesis, and targeting these pathways therapeutically may be a promising strategy for AD treatment.

## Modulation of Mitophagy in AD—A Therapeutic Approach

As impaired mitochondrial function and QC play an important role in AD pathogenesis, pharmacological interventions improving mitochondrial function and QC have been evaluated in various AD models. Along with pharmacological modulation, various lifestyle interventions like intermittent fasting, caloric restriction and vigorous exercise that were shown to induce mitochondrial biogenesis, reduce oxidative stress, and enhance autophagy could also have a beneficial effect on improving mitochondrial health in AD (Halagappa et al., 2007; Mattson, 2015; Nencioni et al., 2018). A recent study by Fang et al. (2019), showed that pharmacological modulation of mitophagy ameliorated Aβ and tau pathology as well as its associated cognitive defects in various AD models. Three potent mitophagy inducers, namely urolithin A (UA), actinonin (AC), and nicotinamide mononucleotide (NMN), identified in a screen, rescued the AD pathology.

UA, a metabolite derived from polyphenol ellagitannins, is reported to be a potent mitophagy inducer in neurons and muscles. In human neuroblastoma SH-SY5Y cells, UA treatment increased the levels of a set of mitophagy-related proteins such as parkin, full-length PINK1 (F-PINK1), p-ULK1 (Ser 555), BECN1, AMBRA1, and Bcl2L13, leading to mitophagy induction. In a *C. elegans* AD model with pan-neuronal expression of Aβ_42_, UA treatment reduced overall Aβ levels and improved memory. This improvement in cognitive function was dependent on the key mitophagy genes *pink-1* and *pdr-1* (mammalian homolog of parkin). The protective role of UA in AD through mitophagy seems to be conserved across species, as an APP/PS1 transgenic mouse model also showed improved learning and memory retention along with a reduction in levels of amyloid peptides Aβ_42_, Aβ_40_ and extracellular Aβ plaques with UA treatment (Fang et al., 2019). UA treatment ameliorated AD pathology by inhibiting phosphorylation of tau in a mitophagy-dependent manner in both *C. elegans* and mice models that recapitulate tau-mediated AD pathology.

AC, a naturally occurring antibacterial agent, was also shown to induce neuronal mitophagy and exhibited similar effects on AD pathology as does UA treatment. The mitophagy induction by AC treatment on a *C. elegans* AD model was dependent on key mitophagy genes *pink-1, pdr-1*, and *dct-1* (mammalian homolog of BNIP3 and BNIP3L). Like UA treatment, AC also improved cognitive ability and mitochondrial health and reduced Aβ plaque burden in AD models, recapitulating both Aβ and tau pathology across species (Fang et al., 2019).

Supplementation with NAD^+^ precursors such as nicotinamide riboside and NMN has been reported to ameliorate pathological features in neurodegenerative disease models like AD and PD (Hou et al., 2019). Nicotinamide riboside treatment showed decreased tau phosphorylation and improved synaptic and cognitive function in two AD mouse models, 3xTgAD mice and 3xTgAD/Polβ^+/–^ (Hou et al., 2018). In a recent study, administration of NMN also showed similar cognitive improvement and AD pathology inhibition in APP/PS1 and 3xTgAD mouse models and in *C. elegans* models, and this was shown to be mediated via mitophagy induction (Fang, 2019; Fang et al., 2019; Lou et al., 2019). As NAD^+^ acts as a cofactor for multiple proteins, such as sirtuins (SIRT 1-7), PARP (poly [ADP-ribose] polymerase), CD38, and SARM1 (sterile alpha and TIR motif-containing 1), all having an important role in the regulation of the autophagy/mitophagy pathway, mitophagy induction using NAD^+^ precursors can be a promising therapeutic approach for treating AD pathology (Maynard et al., 2015; Fang, 2019).

Apart from pharmacological modulation of mitophagy, a genetic approach for mitophagy induction was also shown to be effective in ameliorating AD pathogenesis. Transgenic overexpression of two key mitophagy proteins, PINK1 and parkin, not only improved mitochondrial health through mitophagy induction but also resulted in reduced Aβ levels and AD pathology. Studies showed that gene therapy-mediated overexpression of PINK1 reduced oxidative stress and Aβ levels in an AD mouse model overexpressing mutant APP (Du et al., 2017). This helped alleviate Aβ-induced synaptic dysfunction and cognitive decline. PINK1-mediated mitophagy induction also helped eliminate the mitochondrial pool of Aβ, further contributing to the reduction in total Aβ levels. It was also shown that PINK1 kinase activity is essential for ameliorating AD pathology and cognitive decline in the AD mouse model, as the kinase-deficient PINK1 mutant did not show the protective effect of PINK1 on AD (Du et al., 2017). In addition to PINK1, parkin was also shown to have a role in mediating intraneuronal Aβ clearance. A triple-transgenic AD (3xTg AD) mouse model injected with lentiviral parkin showed increased ubiquitination and clearance of intracellular Aβ aggregates, with a concomitant reduction of extracellular Aβ plaques (Khandelwal et al., 2011). It also stimulated BECN1-dependent autophagy and clearance of damaged mitochondria through mitophagy induction. The parkin-mediated Aβ clearance was also associated with reduced oxidative stress, restored mitochondrial function, improved efficiency of the TCA (tricarboxylic acid) cycle, increased glutamate synthesis, and a re-established neurotransmitter equilibrium, thus making it a prospective candidate for gene therapy against AD pathology (Khandelwal et al., 2011). Further investigations are required to understand the detailed mechanisms underlying the role of PINK1 and parkin in the context of AD. Since genetic modulation can involve ethical considerations, extensive studies regarding aspects of gene therapy are required before considering it as a therapeutic avenue in treating AD.

Apart from removing damaged mitochondria, recent reports suggest that mitophagy also plays a role in alleviating inflammation, that could be used to modulate neuroinflammation in neurodegenerative diseases such as AD. A study by Sliter et al. (2018) showed that the mitophagy-related proteins PINK1 and parkin helped mitigate STING-induced inflammation. The role of mitophagy in alleviating neuroinflammation was further shown in a recent study where induction of mitophagy in microglia, which are phagocytic immune cells in the brain, reduced neuroinflammation and AD pathogenesis (Fang et al., 2019). During AD progression, microglia exhibit increased levels of pro-inflammatory cytokines such as tumor necrosis factor-α (TNF-α) and interleukin-6 (IL6) along with decreased production of anti-inflammatory cytokine IL-10 due to their persistent activated state (Hickman et al., 2008; Lautrup et al., 2019). Additionally, there is activation of the NLR family pyrin domain containing 3 (NLRP3) inflammasome as well as increase in the levels of cleaved caspase-1 in AD models, indicating neuroinflammation (Heneka et al., 2013). Pharmacological induction of mitophagy using UA and AC treatment caused increased expression of microglia-enriched transcriptional regulator IRF7 (interferon regulatory factor 7), engulfment-associated protein CD68, and microglial proliferation marker CD116/CSF2RA, further shifting the microglial population to its phagocytic state. Increased microglial phagocytic activity caused through mitophagy induction promoted enhanced engulfment and removal of Aβ plaques in APP/PS1 AD mice. Along with the enhanced microglial phagocytosis, UA- and AC-induced mitophagy was also shown to increase the levels of anti-inflammatory cytokine IL-10 and to reduce the levels of pro-inflammatory cytokine TNF-α and IL-6 in a PINK1-dependent manner. Mitophagy induction is also associated with decreased activation of the NLRP3 inflammasome, with reduced levels of its downstream effectors such as cleaved caspase-1 and proinflammatory IL-1β in APP/PS1 AD mouse model. These results, combined with the reduction in insoluble Aβ plaques, indicate that pharmacological restoration of mitophagy in microglia increases its phagocytic activity and mitigates NLRP3-dependent neuroinflammation in AD models, thus ameliorating AD pathology (Fang et al., 2019; Lautrup et al., 2019). Therefore, modulation of mitophagy in AD may play a protective role in both neurons and microglia, making it a promising therapeutic target for AD treatment.

## Conclusion

Maintaining mitochondrial integrity is an essential factor that contributes to the effective functioning of a cell. Therefore, any dysfunction in mitochondrial QC pathways can have a detrimental effect on cells like neurons that are critically dependent on mitochondria. Emerging evidence suggest that defects in mitochondria and mitochondrial QC could be one of the primary contributing causes for AD progression. Studies conducted in different AD models across species have revealed that abnormal mitochondrial function, defective mitochondrial dynamics, and compromised mitophagy lead to increased oxidative stress, synaptic dysfunction, neuronal loss, and cognitive decline, thereby contributing to enhanced AD pathology. Owing to the role of mitophagy in AD progression, therapeutic interventions modulating these pathways have been evaluated in different AD models. These studies have shown that mitophagy induction plays a protective role in ameliorating AD pathogenesis by reducing the Aβ plaque burden and neuroinflammation thus delaying cognitive decline. Even though there is significant progress in this field, further research directed towards developing and validating more potent mitophagy inducers is the need of the hour. Eventually, such drug-like mitophagy inducers could be employed as an effective therapeutic strategy against AD pathogenesis.

## Author Contributions

All authors contributed equally to drafting the manuscript. AC contributed by writing the sections “Introduction,” “The Amyloid to Mitochondrial Cascade Hypothesis in the Pathogenesis and Progression of AD,” and “Mitochondrial Dysfunction in AD—A Cause or an Effect?”. CJ wrote the sections “Role of Mitochondrial Quality Control Mechanisms in Maintaining Cellular Homeostasis,” “Impairment of Mitochondrial QC in AD,” “Modulation of Mitophagy in AD—A Therapeutic Approach,” and “Conclusion”. AC created the Figures 1, 2. CJ created the Figure 3. RM contributed to the conceptualization and critical review of the manuscript.

## Conflict of Interest

The authors declare that the research was conducted in the absence of any commercial or financial relationships that could be construed as a potential conflict of interest.
